# Placenta Accreta Spectrum in Mauritania: Epidemiological Characteristics, Clinical Challenges, and Management Perspectives

**DOI:** 10.7759/cureus.93787

**Published:** 2025-10-03

**Authors:** Ahmed Cheikh Mohamed Ahmed, Ali N'Taba, Khadijetou Vilaly, Mohamed Nadi, Telmoudi E Cheikh, Abdellahi Abed, Abdi A Bonahy

**Affiliations:** 1 Obstetrics and Gynecology, Mother and Child Hospital Center of Nouakchott, Nouakchott, MRT; 2 Obstetrics and Gynecology, Faculty of Medicine, Pharmacy and Odontostomatology, University of Nouakchott, Nouakchott, MRT; 3 Radiology, Faculty of Medicine, Pharmacy and Odontostomatology, University of Nouakchott, Nouakchott, MRT

**Keywords:** cesarean delivery, maternal mortality, mauritania, placenta accreta spectrum (pas), postpartum hemorrhage

## Abstract

Background: Placenta accreta spectrum (PAS) represents an increasingly prevalent global health concern, associated with significant maternal morbidity and mortality. The burden is particularly critical in low-income countries. This study reports the experience of Mauritania in the management of PAS.

Methodology: A retrospective cross-sectional observational study was conducted at the Mother-Child Hospital Center of Nouakchott (CHME), the national referral center for gynecological, obstetric, and pediatric pathologies, between January 1, 2023, and December 31, 2024. All cases of PAS diagnosed and managed during this period were included.

Results: A total of 25 cases of PAS were identified among 16,658 deliveries, corresponding to an incidence of 1.50 per 1,000 births (0.15%). The mean maternal age was 35.3 years. The main risk factors observed were previous cesarean section, high multiparity, and advanced maternal age. Blood transfusion was a cornerstone of management. Most patients underwent primary radical surgical treatment with cesarean hysterectomy. Surgical complications represented the main adverse outcomes.

Conclusions: PAS is an emerging and challenging reality in Mauritania. The high prevalence of risk factors and the frequent need for transfusion emphasize the importance of establishing dedicated referral centers. Planned, multidisciplinary, and early management in specialized facilities is crucial to improving maternal and perinatal outcomes.

## Introduction

Placenta accreta spectrum (PAS) represents a group of abnormal placental adherence disorders involving invasion of the myometrium [1]. Three forms are classically distinguished depending on the depth of invasion: placenta accreta, in which chorionic villi superficially invade the myometrium; placenta increta, characterized by deeper penetration without reaching the serosa; and placenta percreta, in which invasion extends through the uterine serosa and may involve adjacent pelvic organs [1].

PAS has become a major public health issue in obstetrics, with incidence rising steadily over the past three decades, from approximately 1 in 4,017 deliveries in the 1980s to 1 in 533 deliveries in the 2000s [2,3]. It is a leading cause of severe postpartum hemorrhage and is associated with maternal mortality rates of up to 30% in the absence of antenatal diagnosis [2,3]. Early recognition of PAS is therefore essential. Antenatal diagnosis enables multidisciplinary care planning and significantly reduces maternal and perinatal morbidity and mortality [4].

Obstetric ultrasound with Doppler remains the first-line diagnostic tool due to its availability and high sensitivity, while magnetic resonance imaging (MRI) serves as a complementary modality for assessing placental invasion and surgical planning [5]. Traditionally, the standard treatment of PAS has been radical management by cesarean hysterectomy. However, recent advances in medical and surgical hemostatic techniques have made conservative management a viable alternative in selected cases, including leaving the placenta in situ, localized (focal) placental/uterine wall resection with uterine repair, uterine or internal iliac (hypogastric) artery ligation, uterine compression sutures, and interventional radiology techniques such as balloon occlusion or embolization. By contrast, adjuvant systemic methotrexate is now used far less often, as routine administration is generally discouraged due to limited proven benefit at term and potential adverse effects. These approaches may preserve fertility and reduce morbidity associated with hysterectomy [6].

The objective of this study was to describe the epidemiological profile of PAS and to evaluate the diagnostic and therapeutic strategies employed at the Mother-Child Hospital Center of Nouakchott (CHME), the national referral center for obstetrics in Mauritania.

## Materials and methods

Type and framework of the study

This is a retrospective cross-sectional observational study conducted in the Gynecology-Obstetrics department of the Mother-Child Hospital Center (CHME) in Nouakchott, a national reference center for gynecologic-obstetric and pediatric pathologies. The study focused on all the files collected between January 1, 2023, and December 31, 2024. The CHME, created in 2009, is the only national reference center in gynecology-obstetrics in Mauritania, receiving patients from all regions of the country. 

Population and sampling

The study population includes all patients who were diagnosed with placenta accreta (in the sense of PAS, including placenta increta or percreta) and those treated at CHME during the study period. The inclusion was exhaustive: no random sampling was carried out, so all eligible patients were retained in the series.

Inclusion criteria

The inclusion criteria selected were as follows: (i) Patients with a clinical and/or pathological diagnosis of placenta accreta (including increta or percreta), during the period from January 1, 2023 to December 31, 2024 and (ii) management carried out at the CHME during said period (patients hospitalized and treated in the institution).

Exclusion criteria

Exclusion criteria mainly included the lack of actionable data. In practice, patients with incomplete medical records (missing essential clinical data or unconfirmed diagnosis) were not included in the study. No other specific exclusion (of the type refusal of care or loss of sight) was encountered, given the retrospective nature of the collection and the acute nature of the pathology studied.

Data collection and statistical analysis

Data Collection

The data were retrospectively collected from available sources, exclusively in paper format. The sources consulted included in particular were as follows: the records of the delivery room at CHME (to identify cases and the outcome of deliveries), patient hospitalization records (containing clinical and surgical reports), the operating records of the obstetrical block (details of surgical interventions), the anesthesia-resuscitation sheets (perioperative information and immediate follow-up), and the anatomopathological examination reports (histological confirmation of the placenta accreta if applicable).

No particular method of quality control was applied during data entry: there was no double entry or systematic cross-checking. Nevertheless, a global review of the collection forms and data entered was carried out to limit transcription errors.

Variables Studied

The main parameters recorded for each patient included: maternal age; history (medical, surgical, and obstetrical, in particular the number of previous cesarean sections, uterine curettages, history of placenta previa, myomectomy, or placenta accreta); clinical data at admission; gestational age at diagnosis and at delivery; diagnostic imaging results; type of surgical management performed; maternal complications occurring intraoperatively and postoperatively; the state of the newborn at birth; whether or not a blood transfusion is required intraoperatively and within 48 hours post-surgery; and finally the immediate operational consequences.

Statistical Analysis

The data analysis was purely descriptive. The results are expressed in absolute and relative frequencies (percentages) for categorical variables, and in central trends (mean, standard deviation, or median depending on the distribution) for relevant quantitative variables. The incidence of placenta accreta over the study period was calculated by relating the number of cases to the total births recorded over the same period. All the data were entered and analyzed using IBM SPSS Statistics for Windows, Version 28 (Released 2021; IBM Corp., Armonk, New York, United States). The tables and graphs were created with Microsoft Excel 2016. No inferential statistical comparison was made, given the non-comparative nature (descriptive study without a control group) of our work.

Ethical considerations

The study protocol received a favorable opinion from the hospital’s ethics committee prior to data collection. Informed consent was not required due to the retrospective design of the study and the use of data already available in the files; however, confidentiality was strictly respected. The information collected has been processed anonymously, and no personal data is disclosed in this report. Furthermore, the authors declare that they have no conflict of interest regarding this study.

## Results

During the study period, 25 cases of placenta accreta were identified among 16,658 deliveries, representing an incidence of 1.50 per 1,000 births (0.15%).

General characteristics of the study population

The mean maternal age was 35.3 years (range: 25-44). A scarred uterus was found in 92% of patients (n=23), most often due to previous cesarean delivery. Uterine curettage was reported in 28% of cases (n=7). No prior history of placenta accreta was observed (Table 1).

**Table 1:** Maternal and Obstetrical Characteristics of the Study Population (N = 25)

Variables	Number (n)	Percentage (%)
Maternal age		
< 30 years	5	20%
30–35 years	8	32%
> 35 years	12	48%
Gravidity, parity		
Primiparous	6	24%
Pauciparous	9	36%
Multiparous	10	40%
History of cesarean sections		
No history	2	8%
Unicicatricial uterus (UUC)	7	28%
Bicicatricial uterus (UBC)	5	20%
Tricicatricial uterus (UTC)	6	24%
> 3 cesarean sections	5	20%
History of curettage	7	28%
History of placenta previa (PP)	18	72%
Placenta previa in the current pregnancy	25	100%
History of myomectomy	0	0%
History of placenta accreta	0	0%

Clinical characteristics

**Figure 1:**
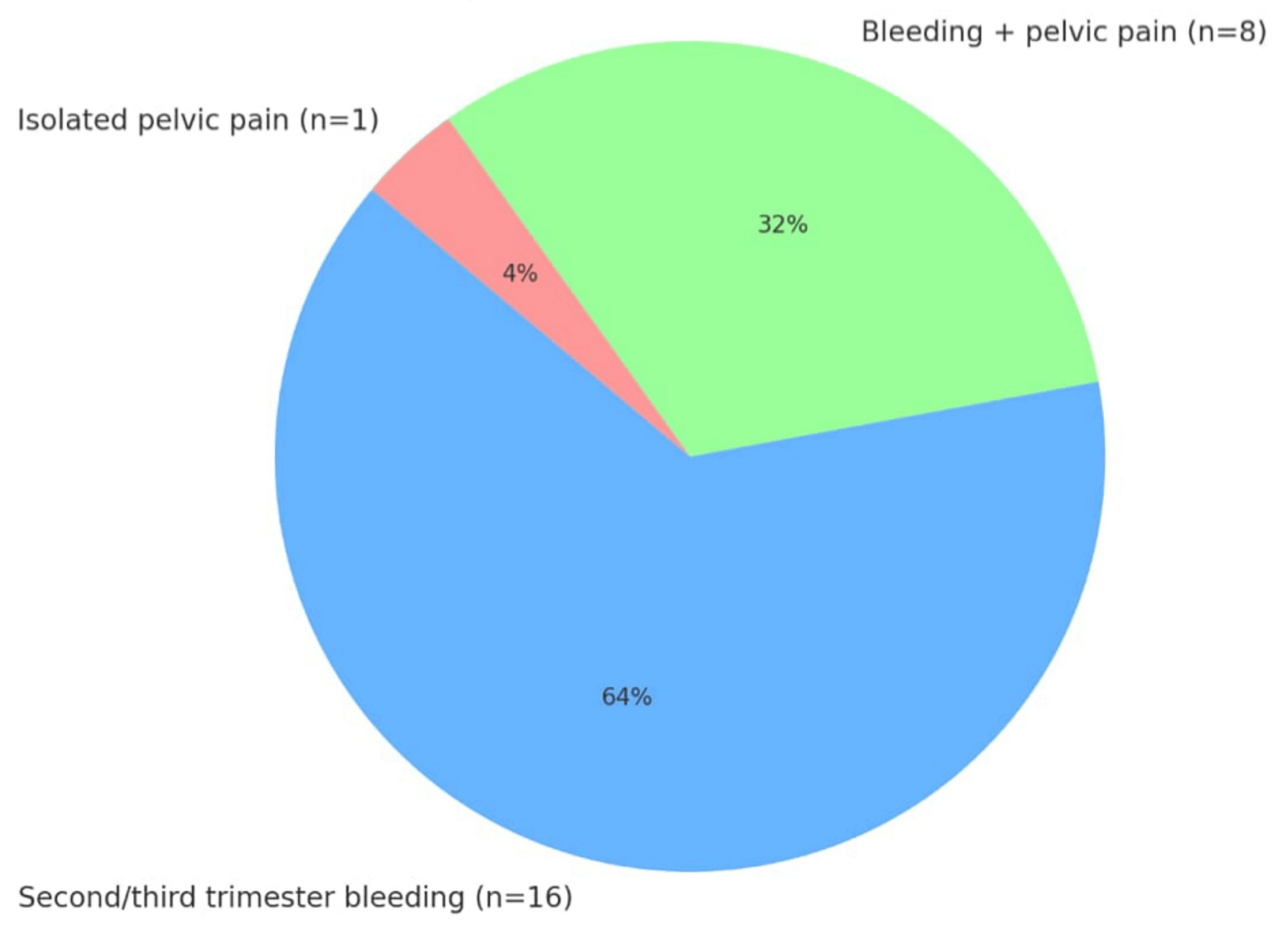
Main Symptoms During Pregnancy (N=25)

Placenta accreta was diagnosed antenatally in 84% of cases (n=21) and incidentally during emergency cesarean in 16% (n=4). Most patients were symptomatic during pregnancy: breakthrough bleeding in the second or third trimester was observed in 64% of cases (n=16), associated with pelvic pain in 32% (n=8). One patient (4%) presented with isolated pelvic pain (Figure 1).

Nearly all women were multiparous (96%), with a median gravidity of 4.7. The mean gestational age at diagnosis was 33 weeks of amenorrhea.

Prenatal ultrasound revealed suggestive signs in 64% of cases (n=16), including placental gaps, abnormal hypervascularization, loss of the retroplacental hypoechogenic zone, and bladder wall irregularities. Placenta previa was universal: anterior insertion in 88% and posterior in 12%. Placenta previa was covering in 40% of cases (n=10), lateral in 36% (n=9), and marginal in 24% (n=6). Magnetic resonance imaging (MRI) was performed in 36% (n=9).

Anatomopathology

Histopathological confirmation was obtained in 60% of cases, contributing to clinical and pathophysiological interpretation.

Management and complications

The mean gestational age at delivery was 34.6 weeks. Twenty-four patients underwent cesarean delivery, while one delivered vaginally. Among cesareans, 64% (n=16) were elective and 32% (n=8) were emergencies. Radical management by primary cesarean-hysterectomy was performed in 84% of cases (n=21). Conservative approaches were attempted in 16% of cases (n=4). In three cases, the placenta was left in situ with favorable outcomes; in one case, local resection of the placental insertion site was required to control bleeding. No additional hemostatic procedures (uterine artery sutures, Bakri balloon, etc.) were reported.

**Figure 2:**
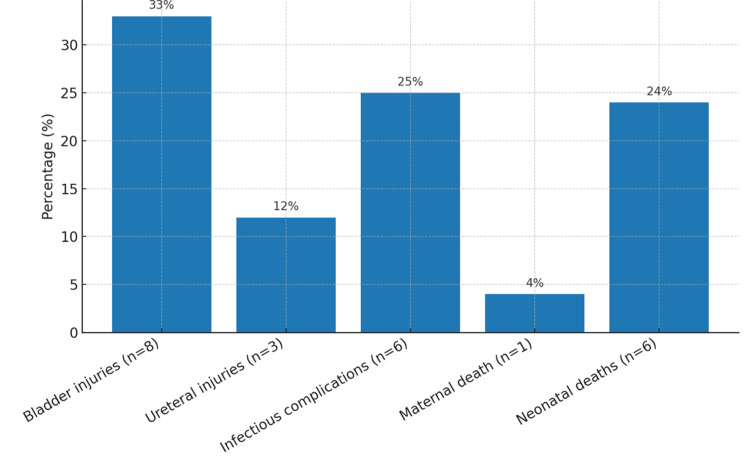
Maternal and Neonatal Complications

The mean postpartum intensive care stay was four days. Blood transfusion was required in 44% of patients (n=11). Surgical complications were dominated by urinary tract injuries: bladder lesions in 33% (n=8) and ureteral injuries in 12% (n=3). Infectious complications (endometritis, wound infection, or sepsis) were recorded in 25% (n=6). The mean Apgar score at 10 minutes was 7.4 (range: 0-10). Maternal mortality was 4% (n=1), and neonatal mortality was 24% (n=6) (Figure 2).

## Discussion

Main results

During the study period, 25 cases of placenta accreta were recorded among 16,658 deliveries, corresponding to an incidence of 1.50 per 1,000 births (0.15%). The main risk factors identified were a history of caesarean section (92%), high multiparity (96%), and previous uterine curettage (28%).

The mean maternal age was 35.3 years (range: 25-44 years), and all cases were associated with placenta previa. Prenatal diagnosis was achieved in 84% of cases, most frequently during the third trimester (mean gestational age: 33 weeks), often prior to the onset of antepartum hemorrhage (64%). The mean gestational age at delivery was 34.6 weeks. Management was radical (caesarean hysterectomy) in 84% of cases and conservative in 16%. Maternal morbidity included blood transfusion in 44% of cases, bladder injury in 33%, ureteric injury in 12%, and infectious complications in 25%. Maternal mortality was 4%, while neonatal mortality reached 24%.

Interpretation of the results

PAS represents an increasingly frequent and potentially life-threatening obstetric complication. Its incidence has risen markedly in recent decades, largely due to the growing prevalence of uterine surgical procedures, particularly caesarean deliveries, that disrupt the integrity of the uterine lining. Approximately three decades ago, Miller et al. [7] reported an incidence of about 1 in 2,500 births. Today, the precise global incidence is difficult to establish, but it is estimated to be around 3 per 1,000 births in the study by Horgan et al. [2].

In our study, we observed 25 cases over a two-year period out of 16,658 deliveries, corresponding to an incidence of 1.50 per 1,000 births (0.15%). This figure is substantially higher than those reported in neighboring countries. For instance, an incidence of 0.017% was reported in Morocco (Slaoui et al.) [8], 0.91% in Egypt (El Gelany et al.) [9], and 0.063% in Jerusalem (Lachman et al.) [10]. This discrepancy may be explained by the central role of our institution: the CHME is the sole referral center in Mauritania with both obstetric intensive care and tertiary-level neonatal services, thereby concentrating the most severe cases referred from across the country.

In our series, the most frequently observed risk factors were consistent with those reported in the literature. Indeed, 92% of our patients had a history of caesarean section, 96% were multigravida or grand multiparous, and the mean maternal age was 35 years. Moreover, all presented with placenta previa accreta (placenta previa associated with accretization) during the current pregnancy. Numerous studies have demonstrated a strong correlation between these factors and the occurrence of placenta accreta. Wu et al., in a case-control study, showed that advancing maternal age increases the risk (OR = 1.13 per year; 95% CI: 1.09-1.19; p < 0.001), as does the presence of at least two previous caesarean sections combined with placenta previa (OR = 51.4; 95% CI: 10.6-248.4; p < 0.0001) [6]. Similarly, a recent meta-analysis by Iacovelli et al. confirmed that advanced maternal age (OR = 3.1; 95% CI: 1.4-7.0) and multiparity (OR = 2.5; 95% CI: 1.7-3.6) are significantly associated with the risk of placenta accreta. This study also showed that, in women with placenta previa and at least one prior caesarean, the risk of placenta accreta is markedly increased compared with women without such a history (OR = 12.0; 95% CI: 1.6-88.0) [11].

Antenatal diagnosis of placenta accreta is a cornerstone of optimal management. It allows the delivery to be planned under the best possible conditions and facilitates the anticipation of necessary resources (multidisciplinary team, blood products, etc.). Several studies confirm that both maternal and perinatal outcomes are significantly improved when PAS is diagnosed prior to delivery (Sugai et al.) [12]. In our center, placenta accreta was suspected antenatally in 84% of cases. Antepartum hemorrhage in the second and third trimesters was the main presenting symptom (about 64% of patients), which explains our relatively high rate of antenatal diagnosis. The mean gestational age at prenatal diagnosis was 33 weeks in our series. These findings are comparable to those reported by Bauwens et al. [13] in France, who observed antepartum hemorrhage as the presenting symptom in 41% of placenta accreta cases. Similarly, a Tunisian study by Bannour et al. (46 cases) found that 36.9% of patients presented with third-trimester bleeding, with a median gestational age at diagnosis of 34 weeks and an antenatal diagnosis rate of 71.7% [14]. These figures are close to those observed in our series.

Obstetric ultrasound remains the reference tool for the prenatal detection of placenta accreta. The addition of color Doppler improves the identification of suggestive signs, such as placental lacunae, utero-placental hypervascularization, interruption of the hypoechogenic interface, and possible extension to adjacent organs. MRI may be used as a second-line modality, either when ultrasound findings are equivocal or to assess locoregional extension in the preoperative setting. Nevertheless, definitive diagnosis can only be established through histopathological examination of the uterine or placental specimen (Berkley; Gielchinsky) [15,16]. In our series, diagnostic confirmation relied largely on intraoperative findings, as histopathological analyses were available in only 60% of cases. This limitation reflects the local context: in Mauritania, there are only two public pathology centers, and obtaining a histological report may take up to two months. Such logistical constraints contribute to loss to follow-up once patients leave the hospital, thereby restricting the systematic collection of pathology results.

International scientific societies recommend that the management of placenta accreta be undertaken in specialized centers with trained multidisciplinary teams. In cases of suspected abnormal placental adherence, referral to such centers is strongly advised. The Royal College of Obstetricians and Gynaecologists (RCOG) recommends scheduling delivery between 35 and 36 weeks of gestation, while the American College of Obstetricians and Gynecologists (ACOG) advocates elective caesarean delivery from 34 weeks (Cahill et al.) [17]. In our series, the mean gestational age at delivery was 34.6 weeks, in line with these recommendations. The high frequency of persistent antepartum hemorrhage in our cohort prompted earlier delivery in order to avoid spontaneous labor and reduce hemorrhagic risk. Importantly, the maternal benefits of planned preterm delivery must always be weighed against the risks of neonatal prematurity.

On this point, the cohort study by Warshak et al. [18] (99 cases) and the analysis by Belfort [19] confirmed that caesarean delivery scheduled between 34 and 35 weeks does not significantly increase neonatal morbidity and represents the optimal management strategy for placenta accreta.

Radical surgical treatment, caesarean section immediately followed by hysterectomy without attempt at uterine preservation, remains the current gold standard for the management of placenta accreta. This was the preferred therapeutic approach in 84% of our cases. Nevertheless, this aggressive strategy is not without risk: hemorrhagic and urological complications, such as bladder and ureteric injuries, are among the most frequent. In the literature, Eller et al. reported massive transfusion rates (≥ 4 units) in 42% of cases, bladder injury in 29%, ureteric lesions in 7%, and infectious complications in 33% during caesarean-hysterectomies for placenta accreta [20]. These figures are comparable to those observed in our series, with transfusion in 44% of patients, bladder injury in 33%, ureteric lesions in 12%, and infectious complications in 25%.

Over the past decade, various uterine-preserving techniques have been described with the dual objective of maintaining patients’ future fertility and potentially reducing maternal morbidity. These conservative approaches, including localized placental resection, arterial embolization, administration of methotrexate, and uterine tamponade, when feasible, have demonstrated a success rate of approximately 80% in certain series (Daney De Marcillac et al.; Sentilly et al.) [21,22]. Several studies also suggest that, when successful, conservative management is associated with lower maternal morbidity and mortality compared to radical treatment.

Nevertheless, these methods are not without significant risks. They expose patients to the possibility of persistent hemorrhage requiring delayed hysterectomy in nearly one-third of cases, prolonged abnormal bleeding (pathological lochia), and infectious complications despite adequate antibiotic prophylaxis (Dimirov et al.) [23]. It therefore appears reasonable to reserve conservative treatment for young women desiring to preserve their reproductive capacity, who accept close and informed follow-up, or in situations where massive placental percreta infiltration into adjacent organs (bladder, bowel, pelvic vascular structures) renders primary hysterectomy particularly high-risk (Su et al.) [24]. Finally, detailed histopathological examination of the surgical specimen remains essential to confirm the diagnosis of placenta accreta and to specify the degree of invasion (accreta, increta, or percreta) [25]. Ideally, each case should undergo histological analysis in order to refine subsequent follow-up, particularly with regard to recurrence risk in future pregnancies. In low-resource settings such as ours, strengthening the capacity of pathology laboratories and reducing turnaround times for histological results represent key areas for improvement in the overall management of PAS.

Implications of the results

In clinical practice, our findings underscore the importance of targeted screening based on enhanced ultrasound surveillance of high-risk patients, complemented by magnetic resonance imaging when diagnostic uncertainty persists. Delivery should be planned in a referral center equipped with an experienced multidisciplinary team and ideally scheduled between 34 and 36 weeks of gestation, in accordance with international recommendations. Radical surgical management remains the reference approach; however, conservative alternatives may be considered in selected young women wishing to preserve their fertility, provided that strict monitoring conditions are ensured.

From a research perspective, it is essential to assess, within our context, the feasibility and safety of conservative strategies as well as their long-term impact on fertility, morbidity, and mortality. Multicenter studies involving several African countries would also be valuable to better estimate the true incidence of placenta accreta and to identify appropriate prevention strategies.

From a public health standpoint, reducing the rate of non-medically indicated caesarean sections should be a priority to limit the incidence of this condition. The development of national protocols for screening and management, together with improved access to timely histopathological examinations, are key measures for optimizing care and ensuring rapid diagnostic confirmation.

Strengths of the study

To the authors' knowledge, this study represents the first documented analysis of placenta accreta in Mauritania. It is based on the exhaustive inclusion of all cases over a defined period and provides detailed data on obstetric, surgical, and neonatal aspects, thereby offering a solid foundation for regional and international comparisons.

Limitations of the study

Several limitations should nevertheless be acknowledged. The retrospective and monocentric design restricts the generalizability of the findings. The reliance on paper-based medical records may have introduced information bias and occasional data loss. Histopathological confirmation was limited by structural and logistical constraints, sometimes leading to substantial delays in obtaining results. Finally, the absence of long-term patient follow-up prevented evaluation of sequelae and subsequent fertility outcomes.

## Conclusions

Our experience at the CHME in Nouakchott confirms that the main risk factors for placenta accreta are prior caesarean delivery, high multiparity, and advanced maternal age. Placenta accreta carries a substantial risk of severe hemorrhage, frequently requiring blood transfusion. In our series, immediate radical surgical management by caesarean hysterectomy was the predominant strategy, with complications mainly related to the surgical procedure itself (bladder and ureteric injuries, infections).

These results underscore the critical need for referral centers dedicated to the management of placenta accreta, where experienced multidisciplinary teams can ensure optimal planning and intervention. Improving antenatal diagnosis, centralizing complex cases, and anticipating resource requirements (blood products, intensive care, interventional radiology) are essential measures to enhance maternal and perinatal outcomes in PAS disorders, both in Mauritania and in other resource-limited settings. Future efforts should also explore conservative treatment strategies.
